# Dynamic Radial MR Imaging for Endoleak Surveillance after Endovascular Repair of Abdominal Aortic Aneurysms with Inconclusive CT Angiography: A Prospective Study

**DOI:** 10.3390/jcm13102913

**Published:** 2024-05-15

**Authors:** Haidara Almansour, Migdat Mustafi, Mario Lescan, Ulrich Grosse, Mateja Andic, Jörg Schmehl, Christoph Artzner, Gerd Grözinger, Sven S. Walter

**Affiliations:** 1 Department for Diagnostic and Interventional Radiology, Eberhard Karls University Tuebingen, University Hospital Tuebingen, 72076 Tuebingen, Germany; haidara.al-mansour@med.uni-tuebingen.de (H.A.); joerg.schmehl@med.uni-tuebingen.de (J.S.); christoph.artzner@diak-stuttgart.de (C.A.); gerd.groezinger@med.uni-tuebingen.de (G.G.); sven.walter@med.uni-tuebingen.de (S.S.W.); 2 Klinik für Thoraxchirurgie-Lungentransplantation und Klinik für Kinderherzchirurgie, Universitätsklinikum des Saarlandes, 66421 Homburg, Germany; migdat.mustafi@uks.eu; 3 Department of Cardiovascular Surgery, University Hospital Freiburg, 79106 Freiburg, Germany; mario.lescan@uniklinik-freiburg.de; 4 Department of Radiology, Cantonal Hospital Frauenfeld, Switzerland Pfaffenholzstrasse 4, 8500 Frauenfeld, Switzerland; 5 Department of Thoracic and Cardiovascular Surgery, University Hospital Tübingen, 72076 Tübingen, Germany; mateja.andic@med.uni-tuebingen.de; 6 Diakonie Klinikum Stuttgart, Department for Radiology, 70176 Stuttgart, Germany

**Keywords:** magnetic resonance angiography, endovascular aortic repair, endoleak, aortic aneurysm, CT angiography

## Abstract

**Background/Objectives**: To assess free-breathing, dynamic radial magnetic resonance angiography (MRA) for detecting endoleaks post-endovascular aortic repair (EVAR) in cases with inconclusive computed tomography angiography (CTA). **Methods**: This prospective single-center study included 17 participants (mean age, 70 ± 9 years; 13 males) who underwent dynamic radial MRI (Golden-angle RAdial Sparse Parallel-Volumetric Interpolated BrEath-hold, GRASP-VIBE) after inconclusive multiphasic CT for the presence of endoleaks during the follow-up of EVAR-treated abdominal aortic aneurysms. CT and MRI datasets were independently assessed by two radiologists for image quality, diagnostic confidence, and the presence/type of endoleak. Statistical analyses included interrater and intermethod agreement, and diagnostic performance (sensitivity, specificity, area under the curve (AUC)). **Results**: Subjective image analysis demonstrated good image quality and interrater agreement (k ≥ 0.6) for both modalities, while diagnostic confidence was significantly higher in MRA (*p* = 0.03). There was significantly improved accuracy for detecting type II endoleaks on MRA (AUC 0.97 [95% CI: 0.87, 1.0]) compared to CTA (AUC 0.66 [95% CI: 0.41, 0.91]; *p* = 0.03). Although MRA demonstrated higher values for sensitivity, specificity, AUC, and interrater agreement, none of the other types nor the overall detection rate for endoleaks showed differences in the diagnostic performance over CT (*p* ≥ 0.12). CTA and MRA revealed slight to moderate intermethod concordance in endoleak detection (k = 0.3–0.64). **Conclusions:** The GRASP-VIBE MRA characterized by high spatial and temporal resolution demonstrates clinical feasibility with good image quality and superior diagnostic confidence. It notably enhances diagnostic performance in detecting and classifying endoleaks, particularly type II, compared to traditional multiphase CTA with inconclusive findings.

## 1. Introduction

Endovascular aortic aneurysm repair (EVAR) is a minimally invasive procedure to treat abdominal aortic aneurysms, utilizing a covered stent-graft to redirect blood flow and stabilize the aneurysm sac [1,2,3]. Contrary to open aortic repair, EVAR necessitates rigorous, lifelong surveillance to monitor stent-graft integrity and to promptly identify relevant complications [3,4,5,6,7].

Endoleaks, characterized by persistent blood flow within the aneurysm sac yet external to the stent-graft lumen, represent the predominant complication following EVAR, occurring in 20–50% of cases [2,8,9,10]. Persistent blood flow to the aneurysm sac due to undetected endoleaks can precipitate the continued expansion of the aneurysm, elevating the risk of eventual rupture [1,7,8,10,11]. This scenario underscores the indispensable role of surveillance for the early detection and timely management of endoleaks, thereby mitigating the risk of aneurysm growth and rupture [1,6,7,8].

Diagnosing and correctly classifying endoleaks poses a diagnostic and therapeutic challenge. Hence, endoleak surveillance requires robust and high-quality follow-up imaging. The Society for Vascular Surgery recommends undergoing imaging within the first 30 days following EVAR and maintaining lifelong surveillance [12]. While Doppler ultrasound offers a non-invasive option for post-EVAR follow-up, triple-phase Computed Tomography Angiography (CTA) is regarded as the standard of care. The triphasic protocol is the favored option, yet a biphasic protocol comprising arterial phase and delayed phase imaging may also be contemplated, particularly in situations where minimizing cumulative radiation exposure is paramount. This approach is preferable to single arterial phase acquisitions. It has been shown that CTA has a sensitivity of up to 92% for endoleak detection, allowing for the detailed assessment of sac expansion and the characterization of endoleak properties [1,13,14,15,16].

Alternatively, postoperative surveillance after EVAR can also be performed using Magnetic Resonance Angiography (MRA), which is already recommended in the event of aneurysmal sac enlargement with uncertain or negative CT results [11,16,17,18]. MRA has not only shown to have comparable sensitivity to CTA, but also outperforms it in the detection of type II endoleaks with a reported sensitivity and specificity of 100%, respectively [6,14,19,20]. Further advantages are time-resolved MRA, the absence of ionizing radiation and iodinated contrast-induced nephropathy, especially in a long-term follow-up [14].

The fairly novel Golden-angle RAdial Sparse Parallel-Volumetric Interpolated BrEath-hold (GRASP-VIBE) technique is a free-breathing MRI sequence for rapid and flexible dynamic contrast-enhanced image acquisition, integrating compressed sensing, parallel imaging, and golden-angle radial sampling to consistently capture volumetric data [21,22,23,24]. With the major advantages of improved spatial and temporal resolution, and a reduced dependency on patients’ respiratory cooperativeness, the efficacy of the GRASP technique has been shown mainly in dynamic liver imaging, head and neck imaging, neuroimaging and four-dimensional MR-angiography [21,22,24,25,26,27,28]. To our best knowledge, this is the first study which examines the application of GRASP-VIBE MRI in the context of ambiguous CTA findings.

The purpose of this prospective study was to assess free-breathing, dynamic radial magnetic resonance angiography for detecting endoleaks post-endovascular aortic repair in cases with inconclusive computed tomography angiography. We hypothesize that GRASP-VIBE is a valuable problem-solving technique that could help guide patient management.

## 2. Materials and Methods

### 2.1. Study Design and Participants

This single-center study was approved by the institutional review board (IRB 166/2018BO2) and written informed consent was obtained from all included participants. Furthermore, the study adhered to the principles outlined in the Declaration of Helsinki and the Health Insurance Portability and Accountability Act.

Recruitment for participants was performed consecutively from April 2018 until July 2023, consisting of the pool of clinical patients seen at the institute’s affiliated department of vascular surgery after internal endovascular aortic repair. Inclusion criteria consisted of (I) inconclusive radiological reporting for the diagnosis of an endoleak in CT, (II) an agreement to participate, and (III) the patients’ ability to be adequately positioned in supine position and the surface coil for the entirety of the exam. Exclusion criteria included patients with (I) external CT exams, (II) more than two months delay between the acquisition of the modalities, (III) severe claustrophobia, and (IV) general contraindications for MRI.

### 2.2. Imaging Protocols

#### 2.2.1. Computed Tomography

Each participant underwent at least biphasic contrast-enhanced abdominal CT using a clinical third-generation dual-source dual energy 2 × 192 slice-CT system (SOMATOM Force, Siemens Healthineers, Forchheim, Germany). Unenhanced CT was not acquired routinely, however, since each portal venous phase at our institute is acquired using dual-energy, virtual unenhanced images were able to be generated if necessary. The scan parameters for arterial phase imaging were as follows: reference tube voltage: 80 kV with CARE kV on; the effective tube current was adapted to the patient’s body mass; quality reference mAs: 200 QrefmAs with CARE Dose 4D on; pitch: 0.8 (or 1.9 if the arterial scan included the thorax); and reconstruction kernel: Bv40. The parameters for the portal venous phase imaging were as follows: dual energy acquisition with reference tube voltage: 100/Sn150 kV with CARE kV on; quality reference mAs: 190/95 mAs with CARE Dose on; pitch: 0.6; and reconstruction kernel Bf40. CT datasets were routinely reconstructed with 3 mm slice thickness.

The intravenous application of contrast agent (Imerone 400 [Iomeprol], Bracco, Konstanz, Germany) was performed using a dual syringe power injector with the volume being adapted to the patients’ bodyweight with a flow rate of at least 3 mL/s, followed by a saline flush. Acquisition of arterial phase was performed using bolus tracking within the abdominal aorta and a threshold of 200 HU with an additional 5-s delay before starting the acquisition. Portal venous phase imaging was started 70 s after injection.

#### 2.2.2. Magnetic Resonance Imaging

Within 2 months of an inconclusive CT reading, each patient underwent a contrast-enhanced MRI using a commercially available clinical 3T scanner (Magnetom Vida, Magnetom Vida Fit; all Siemens Healthineers, Forchheim, Germany) equipped with a commercially available 18-channel surface body coil with a 72-channel spine coil (Siemens Healthineers, Forchheim, Germany). A bolus of contrast agent was administered in all MRI studies adapted to body weight (at least 5 mL Gadobutrol; Gadovist, Bayer Healthcare, Leverkusen, Germany) with a flow rate of 1.5 mL/s followed by a saline flush of 20 mL. In addition to the institute’s standard protocol, a dynamic GRASP-VIBE sequence was acquired; the acquisition parameters are presented in Table 1.

### 2.3. Image Analysis

The imaging datasets of each patient were first pseudonymized. Independent analysis was performed in a randomized and blinded fashion by two board-certified radiologists with 5 years (H.A.) and 8 years (S.S.W.) of experience in vascular imaging. To reduce recall bias, all cases were modality-based assessed in two independent sessions 6 weeks apart. The evaluation was performed in a certified reading room, equipped with a diagnostic-grade 32″ display and an enterprise-grade image archiving and communication system (Syngo.Via Enterprise Browser Version 2.3.1, Siemens Healthcare, Erlangen, Germany). Each reader employed their individual reading settings, including custom window and level settings, and magnifications.

### 2.4. Outcome Variables

#### 2.4.1. Image Quality Analysis

For internal quality control, an analysis of general image quality was performed consisting of motion, artifacts, noise, and overall image quality for CT and MRI, respectively. Additionally, edge sharpness, contrast resolution, fat suppression, and partial volume effect were assessed for MRI. Each outcome variable was assessed using a 5-point Likert scale with equidistant intervals (1 = very good/none; 2 = good/mild; 3 = adequate; 4 = bad/severe; 5 = very bad). The diagnostic confidence for the presence or absence of an endoleak for was also assessed on an equidistant 5-point Likert scale for both modalities (1 = highly confident; 2 = confident; 3 = moderately confident; 4 = low confidence; 5 = not confident).

#### 2.4.2. Detection/Classification of Endoleaks

The imaging datasets were evaluated regarding the presence or absence of endoleaks. In case of a present endoleak, the observer classified the type of the endoleak according to the Cardiovascular and Interventional Radiological Society of Europe (CIRSE) guidelines into five types: (I) endoleak on the attachment side of the endovascular prosthesis (Ia: proximal end; Ib: distal end), (II) retrograde flow via smaller branch vessels, (III) mechanical failure of the implanted graft, (IV) graft porosity, and (V) enlargement of the aneurysm sack without visualized endoleak [29]. Finally, MRI exams of type II endoleaks were additionally assessed on the possibility of identifying inflow and outflow vessels (1 = flow direction assessable, 2 = flow direction not assessable).

The reference standard comprised a comprehensive combination of clinical, radiological, and long-term follow-up data until February 2024. A senior interventional radiologist (G.G.), with 13 years of experience in vascular imaging and treatment, who was independent from the initial readouts, had full access to the complete case file, encompassing clinical reports and imaging datasets from EVAR implantation to the latest patient follow-up. Following a thorough examination of these components, a determination regarding the presence and classification of endoleaks was reached.

### 2.5. Statistical Analysis

Statistical analyses and computations were conducted using SPSS (IBM SPSS Statistics 29, Armonk, NY, USA). Variables are displayed as mean ± standard deviation or as the number of participants with a percentage. Likert-scale grading of image quality parameters is given as a median value with minimum, first to third quartiles, and maximum. Differences in image quality assessments between raters were rigorously analyzed using the Wilcoxon signed-rank test. The interrater and intermethod agreements were quantified using Kendall’s Tau (image quality) and Cohen’s kappa. Sensitivity, specificity, and areas under the receiver operating characteristic curve (AUCs) were used to calculate the diagnostic performance using the reference standard described above. *p* < 0.05 was considered statistically significant.

## 3. Results

### 3.1. Participant Characteristics

**Figure 1:**
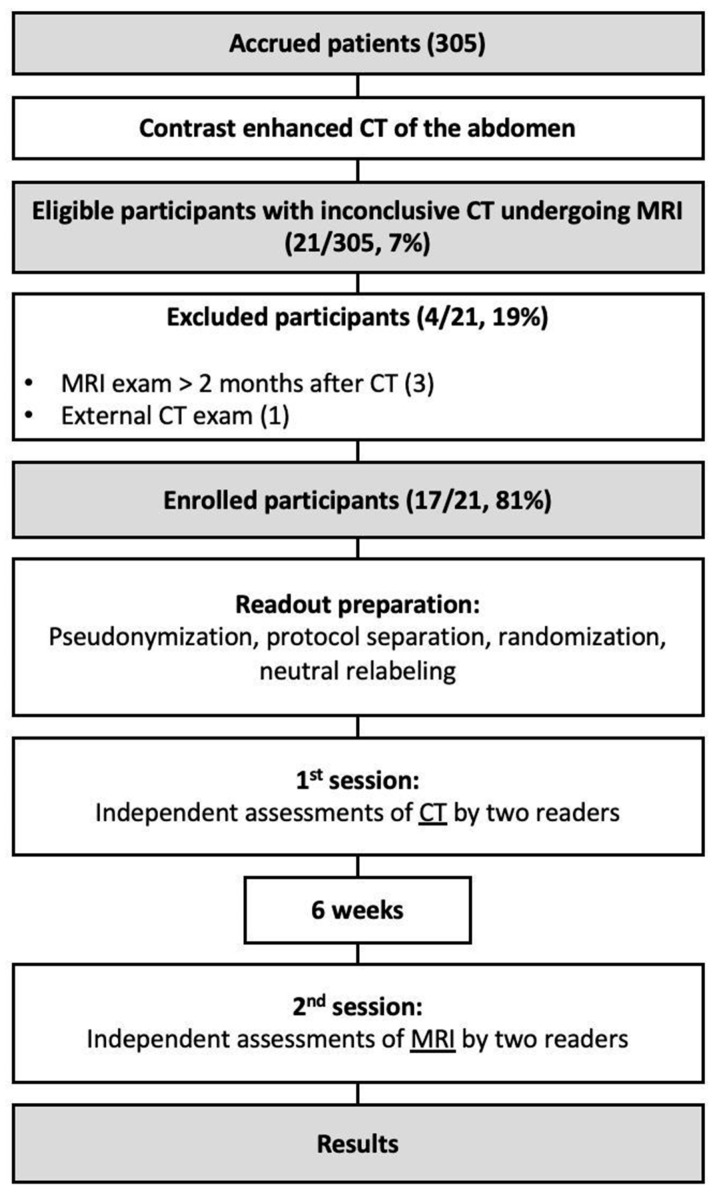
Flow diagram illustrating the inclusion and exclusion of the study’s participants.

During the recruiting phase there were 305 eligible participants, while 17 (6%) were enrolled into the study (70 ± 9 years). Participant selection and exclusion criteria are illustrated in Figure 1. The cohort consisted predominantly of male patients with an elevated BMI. Each of the implemented stent graft was nitinol based. In seven patients (41%), the MRI resulted in a change in management with interventional treatment of the endoleak (type Ia: 1, type Ib: 2; type II: 4). Patients of the cohort were followed up for a median of 31 months after they underwent the CT exam with inconclusive results. Participant characteristics are summarized in Table 2.

### 3.2. Technical Analysis

For CT motion, artifacts were predominantly absent. Artifacts, image noise, and overall image quality were rated as good by both readers without significant difference, respectively. There was good interrater agreement for each of the assessed image parameters for CT (k ≥ 0.68) (Table 3).

In addition to predominantly absent motion artifacts, MRI datasets also demonstrated very good fat suppression. The remaining image parameters were rated as good with good interrater agreement (k ≥ 0.6). None of the assessed parameters demonstrated a significant difference between the raters (Table 3).

The median diagnostic confidence was rated good and very good for CT and MRI, respectively, with MRI achieving a significantly higher confidence for the diagnosis of endoleaks (*p* = 0.03).

### 3.3. Frequency of Endoleaks and Intermethod Concordance

Table 4 summarizes the frequencies on the presence and type of endoleaks in CT and MRI as assessed by the individual raters. The detection rates of endoleaks and type of endoleak had slight to moderate intermethod concordance (k = 0.3–0.64) between the CT and MRI exam.

### 3.4. Diagnostic Performance

The diagnostic performance is summarized in Table 5. Although MRI GRASP-VIBE demonstrated higher diagnostic accuracy and interrater agreement (CT: 0.38 [95% CI: 0.0, 0.76); MRI: 0.74 [95% CI: 0.39, 1.0]), there was no significant difference in the overall diagnostic performance over CT (*p* = 0.12).

**Figure 2:**
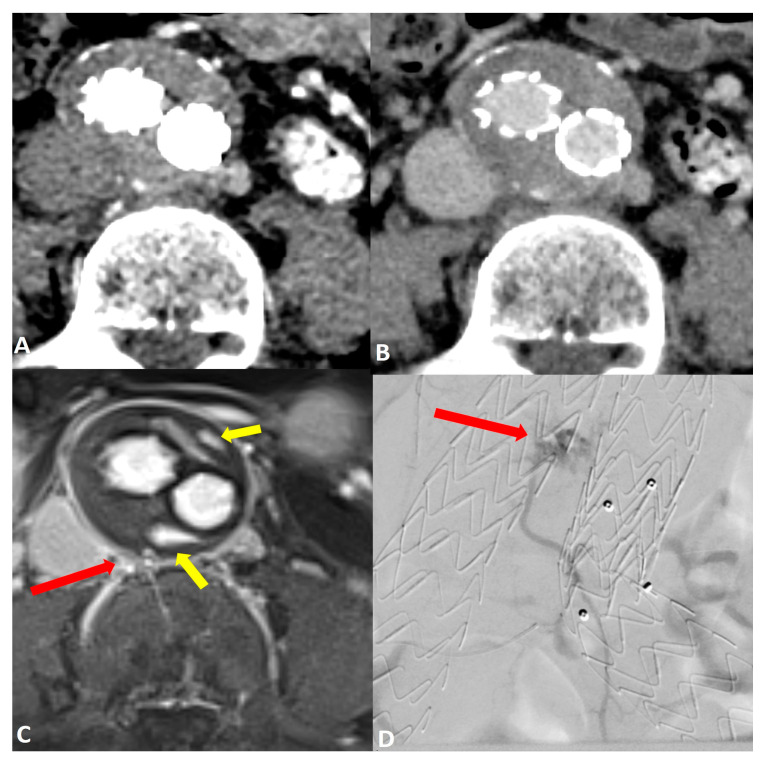
Post-operative imaging of a 57-year-old woman who underwent endovascular aneurysm repair (EVAR) of the abdominal aorta. Axial CT angiograms in (**A**) the post-contrast arterial phase and (**B**) the delayed phase did not reveal a clear endoleak within the growing aneurysm sac. Axial dynamic imaging utilizing the GRASP-VIBE sequence (**C**) revealed a type II endoleak with the right iliolumbar artery directly communicating with the aneurysm sac, denoted by a red arrow. Notably, there was an accumulation of contrast material at the anterior and posterior peripheral aspects of the aneurysm sac, marked by yellow arrows. This endoleak was subsequently confirmed in the digital subtraction angiography ((**D**), red arrow) and was promptly treated.

**Figure 3:**
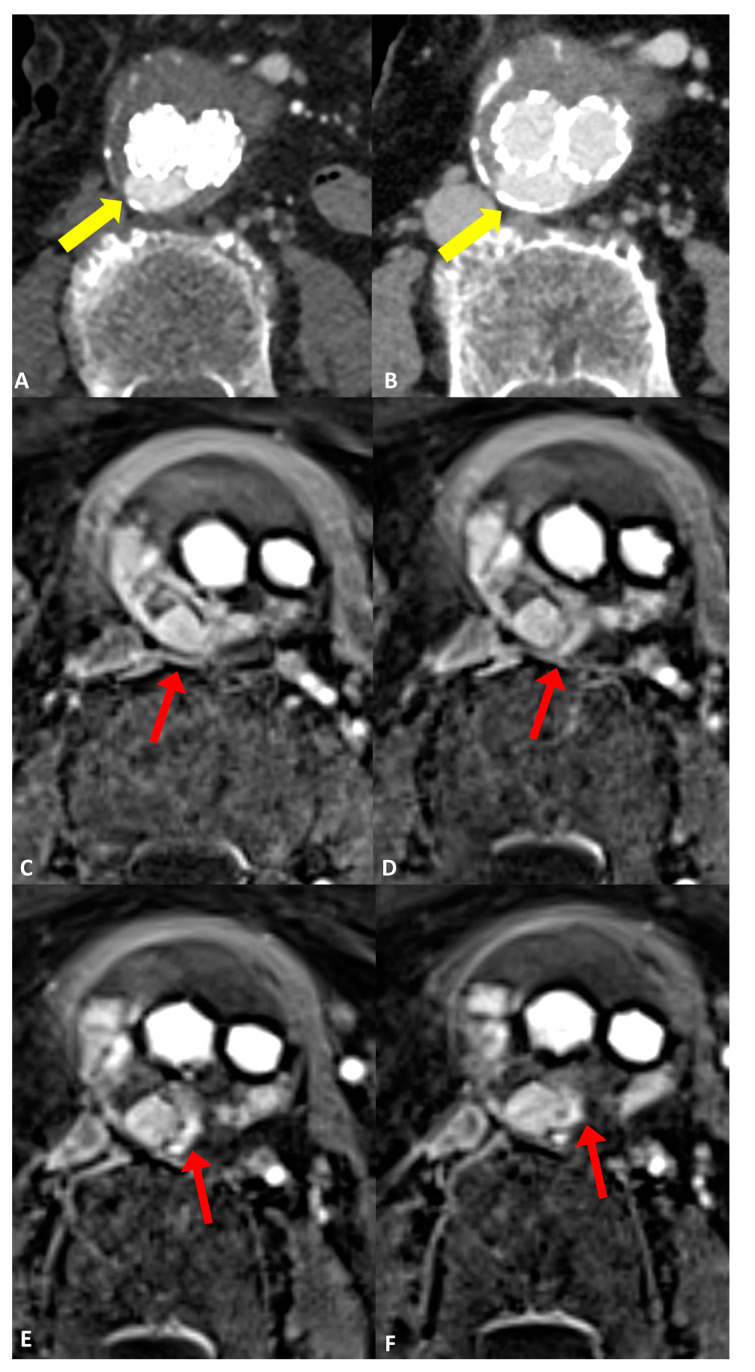
Follow-up imaging after endovascular aortic aneurysm repair (EVAR) in a 79-year-old woman diagnosed with an infrarenal abdominal aortic aneurysm. Axial CT angiograms revealed an endoleak with inconclusive classification (type I, II, or III), marked by yellow arrows in (**A**) the post-contrast arterial phase and (**B**) the post-contrast delayed phase. (**C**–**F**) four consecutive axial slices utilizing the dynamic GRASP-VIBE sequence illustrated a communication between the right lumbar artery and the sac, highlighted by a red arrow in (**C**), followed by the entry of contrast material into the aneurysm sac and its distribution within, evident in (**D**–**F**) with red arrows. Notably, the contrast material displayed a similar signal to that of the right lumbar artery, which was less pronounced compared to the aorta/common iliac arteries. This observation favors the diagnosis of a type II endoleak originating from the right lumbar artery, as opposed to a graft defect.

Comparing the endoleak types, there was significantly improved diagnostic accuracy for the detection of type II endoleaks in MRI (AUC 0.97 [95% CI: 0.87, 1.0]) compared to CT (AUC 0.66 [95% CI: 0.41, 0.91]; *p* = 0.03) (Figure 2). Sensitivity and specificity were also increased in MRI GRASP-VRIBE compared to CT with 100%/91% and 50%/82%, respectively. Moreover, the capability of dynamic contrast-enhanced MRA to identify the inflow and outflow vessels associated with type II endoleaks was rated as highly feasible by both raters, with no statistically significant differences observed between their assessments (*p* = 0.37) (Figure 3).

MRI demonstrated an improved diagnostic performance for type I endoleaks, especially type Ib (CT: AUC 0.68 [95% CI: 0.19, 1.0]; MRI: AUC 1.0 [95% CI: 1.0, 1.0]) while type Ia had similar accuracy. However, there was no significant difference between CT and MRI GRASP-VIBE (*p* > 0.12).

Both modalities had a similar diagnostic performance for the detection of type III endoleaks with an AUC of 0.97 [95% CI: 0.79, 1.0] and 1.0 [95% CI: 1.0, 1.0] in CT and MRI, respectively. Sensitivity and specificity ranged between 94% and 100%.

Interrater agreement was consistently higher for MRI GRASP-VIBE, except for type III endoleaks with an equal agreement (Table 5).

## 4. Discussion

The accurate detection of endoleaks in previously inclusive CTA, using a rapid and free-breathing MRI sequence with flexible dynamic contrast, can add value through improving early management of complications following EVAR implantation. This study investigated the performance of such contrast-enhanced dynamic compressed sensed radial MRA (GRASP-VIBE) in patients with inconclusive multiphasic CT for the presence of endoleaks during the follow up of EVAR-treated abdominal aortic aneurysms. We found that the free-breathing GRASP-VIBE enabled a reliable and robust acquisition of image datasets with good quality and significantly increased diagnostic confidence compared to multiphase CTA. Furthermore, the detection of type II endoleaks was significantly improved on GRASP-VIBE sequences compared to CTA. Although MRA demonstrated improved sensitivity, specificity, AUC, and interrater agreement, none of the other types nor the overall detection for endoleaks demonstrated a significant difference in the diagnostic performance over CTA.

Previous approaches of using MR to detect endoleaks included repetitive use of non-contrast-enhanced MR of the aorta to detect blood products using T1-weighted sequences or by using T1-volume interpolated breath hold examination (VIBE) in the delayed phase [17,30]. In a systematic review examining the sensitivity of MRA in comparison to CTA in detecting endoleaks, a total of eleven studies were included, encompassing 369 patients with 562 MRI and 562 CTA examinations. The results showed that MRI revealed significantly more endoleaks (n = 132) after EVAR than CT [17]. The authors concluded that MRI has higher sensitivity than CTA in identifying post-EVAR endoleaks, particularly type II endoleaks [17]. This is similar to our results with sensitivities for CT and MRA ranging between 50–100% and 100%, and the specificities ranging from 57 to 94% and from 86 to 100% depending on the type of endoleak, respectively. The review explained this with the superior soft-tissue contrast and by enhanced contrast sensitivity for minute and slow-flowing endoleaks. Therefore, the authors recommended that MRI should be performed in suspicious cases despite negative or inconclusive CTA results. However, these 11 studies only provided static information and included T1-weighted post contrast imaging and none of the studies utilized time-resolved dynamic MRA. Hence, no assessment of contrast dynamics was performed and small endoleaks might have been missed [17].

In this context, time-resolved MRA is a valuable imaging technique [17,31,32,33]. For instance, an early study by Cohen et al. compared time-resolved MRA with digital subtraction angiography (DSA), in the classification of endoleaks in thirty-one patients after EVAR. The authors showed an agreement between the two methods in 97% of cases [31]. In our study, the agreement between CTA and dynamic radial MRA for endoleaks and the respective types demonstrated only moderate concordance with k-values ranging from 0.3 to 0.64. The relatively low concordance is most likely due to the superior diagnostic performance of the GRASP-VIBE sequence, although only significant for type II endoleaks, and an elevated number of false-positive and false-negative findings in non-dynamic CT.

Pathologies of the aorta often have dynamic components, thus the imaging techniques utilized to capture these pathologies should also be dynamic, flexible, fast and robust against motion artifacts [34]. Adding time as another dimension for assessing the in- and outflow of contrast in the aorta is helpful to not only better understand aortic pathologies but also improve surveillance and clinical decision making. In this context, radial sampling has several advantages over conventional (cartesian) sampling, with enhanced image quality and reduced artifacts. This could partially be achieved, due to oversampling of the center of k-space and data redundancy, which can be utilized to correct motion artifacts [22]. Moreover, its high temporal and spatial resolution and image quality not only facilitated the precise detection and classification of endoleaks but also enabled direct visualization of the inflow and outflow arteries, particularly in cases of type II endoleaks. This holds clinical significance, particularly in guiding treatment decisions, given that type II endoleaks often necessitate intervention in patients with enlarging aneurysm sacs as per the current American and European guidelines [12,13,35]. Additionally, these results are of clinical interest as the utilization of this technique resulted in a change in management in 41% of cases in our study.

Our study has several limitations. Firstly, the small sample size compromises the external validity of these findings. This small sample size can be explained by the fact that only patients with inconclusive findings in the CTA were included. For most patients and most cases, there is no need for additional imaging when the findings are conclusive as additional imaging would either be unnecessary or delay treatment. Hence, in this research project, we only included patients who needed additional imaging (MRI) as a problem-solving tool to guide their management. Furthermore, in most patients included in our study, the mean time difference between CTA and MRA was less than one month. However, in five patients, the duration between the two modalities was over one month. This is a limiting factor, because a prolonged duration between CTA and MRI could augment the identification of supplementary endoleaks via MRI, as new endoleaks might have emerged or existing ones could have intensified in size or severity during this timeframe. However, retrospectively, the included patients with 1–2 months between the modalities remained consistent. Secondly, this study exclusively examined scans conducted at 3 Tesla using a singular high-relaxivity contrast agent, gadobutrol. Employing lower field strengths or different contrast agents with lesser relaxation enhancement may significantly affect angiographic quality. Thirdly, our reference standard was a combined radiological and clinical assessment with a long follow-up. In our study, confirmation using an unbiased reference standard such as DSA was not consistently conducted. Nonetheless, it is worth noting that DSA might also overlook endoleaks, particularly in cases where non-selective DSA is employed [17,31]. Lastly, susceptibility artifacts caused by clips or older stainless-steel stent-grafts may impede MRI evaluation of patients post-EVAR, which was the case in one patient in our study cohort. Nevertheless, this prospective study serves as a preliminary investigation and a proof-of-concept of this dynamic GRASP-VIBE sequence and provides encouraging results for further clinical application.

## 5. Conclusions

In conclusion, the GRASP-VIBE MRA characterized by high spatial and temporal resolution, demonstrates clinical feasibility with good image quality and superior diagnostic confidence. It notably enhances diagnostic performance in detecting and classifying endoleaks, particularly type II, compared to traditional multiphase CTA with inconclusive findings. A potential and important clinical application for this technique is in patients after EVAR without a visible endoleak in CTA, yet with a growing aneurysm sac. This technique could serve as a problem-solving tool for further improving diagnostic performance and endoleak conspicuity with an acceptable acquisition time and might prove indispensable in guiding clinical management. Further prospective studies comparing GRASP-VIBE sequences with conventional time-resolved MRA and larger participant samples are warranted to validate the findings of this proof-of-principle study.

## Figures and Tables

**Table 1:** Acquisition parameters for the dynamic GRASP-VIBE MRI sequence.

Parameter	Value
Acquisition time (min:s)	2:54
Temporal Resolution (s)	3.1
Voxel Size (mm^3^)	0.9 × 0.9 × 3.0
Number of Excitations/Number of Signal Averages	1
Orientation	Axial
Slice Thickness (mm)	3
Number of slices per slab	72
Repetition Time (ms)	3
Echo Time (ms)	1.57
Flip angle (°)	15
Bandwidth (Hz/Px)	980
Acceleration	Compressed sensing

Note: Hz: Hertz, mm: millimeter, ms: millisecond, min: minute, s: second, Px: pixel.

**Table 2:** Demographic characteristics of the cohort.

Characteristics	n = 17
Age (y)	70 ± 9
Sex *	
Female	4 (23)
Male	13 (77)
BMI	27 ± 3
GFR at CT (mL/min/1.73 m^2^)	72 ± 19
Postoperative EVAR-related Symptoms *	
Yes	2 (12)
No	15 (88)
CT Phase Acquisition *	
Biphasic CTA	11 (65)
Triple phase CTA	6 (35)
Radiation Dose (DLP in mGycm)	907.9 ± 374.2
Nitinol-based Stent Graft *	17 (100)
Time from EVAR to Inconclusive CT (m)	16 ± 30
Time Between Modalities (d)	26 ± 23
Patients Undergoing Interventional Treatment	7 (41)
Last Follow-Up Since Inconclusive CT (m)	31 ± 26

Note: Unless otherwise specified, data is given in mean ± standard deviation. * Data is given in number of participants with percentage in parentheses. BMI: body mass index, CTA: computed tomography angiography, d: days, DLP: dose length product, EVAR: endovascular aortic repair, GFR: glomerular filtration rate, m: months, y: years.

**Table 3:** Ratings of image quality parameters by the raters for CT and MRI GRASP-VIBE sequence.

Modality	CT				MRI			
	Rater 1 *	Rater 2 *	*p*	Interrater Agreement ^†^	Rater 1 *	Rater 2 *	*p*	Interrater Agreement ^†^
Motion	1 (1, 1, 1, 2)	1 (1, 1, 1, 2)	0.32	0.69 (0.45, 0.83)	1 (1, 1, 2, 2)	1 (1, 1, 1, 2)	0.16	0.72 (0.50, 0.85
Artifacts	2 (1, 2, 2, 3)	2 (1, 1, 2, 3)	0.66	0.68 (0.44, 0.83)	2 (1, 2, 3, 4)	2 (1, 2, 3, 4)	0.66	0.76 (0.56, 0.87)
Image Noise	2 (1, 2, 3, 3)	2 (2, 2, 3, 4)	0.32	0.71 (0.48, 0.85)	2 (1, 2, 2, 4)	2 (1, 2, 2, 3)	0.08	0.83 (0.68, 0.91)
Edge Sharpness	-	-	-	-	2 (1, 2, 2, 3)	2 (1, 1, 2, 3)		0.63 (0.37, 0.80)
Contrast Resolution	-	-	-	-	2 (1, 2, 3, 3)	2 (1, 2, 2, 3)	0.32	0.70 (0.47, 0.84)
Fat Suppression	-	-	-	-	1 (1, 1, 1, 2)	1 (1, 1, 1, 2)	>0.99	1.0 (1.0, 1.0)
Partial Volume Effect	-	-	-	-	2 (1, 2, 2, 3)	2 (1, 1, 2, 2)	0.6	0.60 (0.32, 0.78)
Overall Image Quality	2 (1, 1, 2, 2)	2 (1, 1, 2, 3)	0.16	0.82 (0.66, 0.81)	2 (1, 2, 2, 3)	2 (1, 2, 2, 3)	0.08	0.72 (0.50, 0.85)
Diagnostic Confidence	2 (1, 2, 2, 3)	2 (1, 1, 3, 4)	0.41	0.70 (0.48, 0.92)	1 (1, 1, 2, 3)	1 (1, 1, 2, 3)	0.32	0.69 (0.45, 0.84)

Note: Values are given as median value with the minimum, first to third quartiles, and maximum. * Based on 5-point Likert scale (1 = very good; 5 = very bad). ^†^ Data are k values with 95% confidence interval in parenthesis.

**Table 4:** Rater assessment for the presence and type of endoleak with CT and MRI GRASP-VIBE.

Abnormality *	Reference Standard ^†^	Rater 1			Rater 2		
		Frequency (n = 17) ^†^		Intermethod Agreement ^‡^	Frequency (n = 17) ^†^		Intermethod Agreement ^‡^
		CT	MRI		CT	MRI	
Endoleak	10 (59)	10 (59)	11 (65)	0.38 (0.0, 0.76)	12 (71)	11 (65)	0.23 (0.0, 0.68)
Type I	3 (18)	5 (29)	3 (18)	0.36 (0.0, 0.82)	5 (29)	3 (18)	0.36 (0.0, 0.85)
Ia	1 (6)	2 (12)	1 (6)	0.64 (0.17, 1.0)	2 (12)	1 (6)	0.64 (0.17, 1.0)
Ib	2 (12)	3 (18)	2 (12)	0.3 (0.0, 0.89)	3 (18)	2 (12)	0.3 (0.0, 0.89)
Type II	6 (35)	4 (24)	6 (35)	0.44 (0.0, 0.89)	5 (29)	7 (41)	0.24 (0.0, 0.7)
Type III	1 (6)	1 (6)	2 (12)	0.64 (0.17, 1.0)	2 (12)	1 (6)	0.64 (0.17, 1.0)

* No type IV or V endoleaks were seen. ^†^ Data in parenthesis are percentages. ^‡^ Data are k values with 95% confidence interval in parenthesis.

**Table 5:** Diagnostic performance.

Abnormality ** and Modality	Interrater Agreement ^‡^	No. of Findings *				Sensitivity (%)	Specificity (%)	AUC	*p*
		TP	FP	TN	FN				
Endoleak									0.12
CT	0.38 (0.0, 0.76)	9	3	3	2	82 (48, 98)	50 (12, 88)	0.69 (0.45, 0.92)	
MRI	0.74 (0.39, 1.0)	10	1	6	0	100 (69, 100)	86 (42, 99)	0.93 (0.79, 1.0)	
EL Type I									0.12
CT	0.43 (0.03, 0.83)	2	3	11	1	67 (9, 99)	79 (49, 95)	0.73 (0.38, 1.0)	
MRI	1.0 (1.0, 1.0)	3	0	14	0	100 (29, 100)	100 (77, 100)	1.0 (1.0, 1.0)	
Type Ia									>0.99
CT	0.43 (0.03, 0.83)	1	1	15	0	100 (3, 100)	94 (70, 100)	0.97 (0.91, 1.0)	
MRI	1.0 (1.0, 1.0)	1	0	16	0	100 (3, 100)	100 (79, 100)	1.0 (1.0, 1.0)	
Type Ib									0.21
CT	0.60 (0.09, 1.0)	1	2	13	1	50 (2, 99)	87 (60, 98)	0.68 (0.19, 1.0)	
MRI	1.0 (1.0, 1.0)	2	0	15	0	100 (16, 100)	100 (78, 100)	1.0 (1.0, 1.0)	
EL Type II									0.03
CT	0.85 (0.57, 1.0)	3	2	9	3	50 (12, 88)	82 (48, 98)	0.66 (0.41, 0.91)	
MRI	0.88 (0.64, 1.0)	6	1	10	0	100 (54, 100)	91 (59, 1.0)	0.96 (0.87, 1.0	
EL Type III									>0.99
CT	0.64 (0.17, 1.0)	1	1	15	0	100 (3, 100)	94 (70, 100)	0.97 (0.79, 1.0)	
MRI	0.64 (0.17, 1.0)	1	0	16	0	100 (3, 100)	100 (79, 100)	1.0 (1.0, 1.0)	

Note: Data in parenthesis are 95% confidence intervals. AUC: area under the receiver operating characteristic curve, EL: endoleak, FN: false-negative findings, FP: false-positive findings, TN: true-negative findings, TP: true-positive findings. * Data are given in number of patients (n = 17). ** No type IV or V endoleaks were seen. ^‡^ Data are k values.

## Data Availability

Data can be obtained from the corresponding author upon reasonable request.
